# Key factors affecting health promoting behaviors among adolescents: a scoping review

**DOI:** 10.1186/s12913-023-10510-x

**Published:** 2024-01-11

**Authors:** Jafar Sadegh Tabrizi, Leila Doshmangir, Najibeh Khoshmaram, Elham Shakibazadeh, Hosein Mashhadi Abdolahi, Roghayeh Khabiri

**Affiliations:** 1 Tabriz Health Services Management Research Center, Health Management and Safety Promotion, Tabriz University of Medical Sciences, Tabriz, Iran; 2 Social Determinants of Health Research Center, Health Management and Safety Promotion Research Institute, Tabriz University of Medical Sciences, Tabriz, Iran; 3 Student Research Committee, Tabriz University of Medical Sciences, Tabriz, Iran; 4 Department of Health Education and Promotion, School of Public Health, Tehran University of Medical Sciences, Tehran, Iran

**Keywords:** Adolescent, Health promotion, Behavior, Scoping review

## Abstract

**Background:**

Health-promoting behaviors have been noticed recently as one of the most critical factors in raising life expectancy, which can be formed during adolescence. Thus, the current scoping review aimed to identify the key factors affecting health-promoting behaviors among adolescents.

**Methods:**

In this scoping review, we searched multiple English online databases, including PubMed, Web of Science, Science Direct, ProQuest, and Scopus, for articles published between 1977 and 2020. All eligible studies describing health-promoting behaviors in adolescents were included. We followed the JBI guideline for conducting a scoping review and increasing the study’s rigor. Extracted data were synthesized through inductive approaches.

**Results:**

A total of 3199 articles were identified during the first phase. After the screening process, 20 articles were found eligible for final inclusion. Educational factors (individualized education and school health promotion programs), Economic factors (income, economic incentives and national health insurance), Social factors (support system, responsibility and peers), Spiritual, Psychological and Personal factors (gender, family structure, patterns of living, and medical problems) were found effective in health-promoting behaviors among adolescents.

**Conclusions:**

Health-promoting behaviors among adolescents require careful consideration. The current review identified some fundamental factors affecting health-promoting behaviors in adolescents. Based on the findings, it is recommended that policymakers and healthcare providers develop several interventions based on identified factors to increase adolescent’s health-promoting behaviors among adolescents.

**Supplementary Information:**

The online version contains supplementary material available at 10.1186/s12913-023-10510-x.

## Background

Adolescence is a period of significant transformations and discoveries. It is a time to affirm one’s personality and develop deeper relations within society, school environment, and family [1]. Adolescents may experience physical changes, increasing aggressiveness, rebellious behaviors, and difficulty communicating appropriately [2].

Unhealthy behaviors such as smoking, unhealthy diet, risky sexual behaviors, and sedentary lifestyle usually start in this period of life. They can finally lead to the onset of chronic diseases in adulthood [3–5]. Chronic diseases or non-communicable diseases (NCDs) have been assigned the first rank of deaths in all ages globally [6]. It is estimated that 50% of individuals aged 65 years and above suffer from at least one NCD [7, 8]. According to the World Health Organization (WHO), NCDs cause the death of 38 million people annually in the world, with almost three-quarters in low- or middle-income countries [6]. The NCDs ' behavioral and biological risk factors are formed in childhood and adolescence and remain until adulthood [9–11].

Health-promoting behaviors have been considered a basic way to prevent chronic diseases [7, 8, 12]. The most important health-promoting behaviors include health responsibility, exercise, nutrition, social support, stress management and life appreciation [13–15]. Health-promoting behaviors raise life expectancy by improving lifestyle and decreasing healthcare costs [16, 17]. Research studies have shown that 53% of deaths are related to hazardous lifestyles and unhealthy behaviors [18].

A healthy lifestyle improves the quality of life and reduces illness and disability [19]. However, adolescents confront many obstacles in employing health-promoting behaviors. These obstacles include poor social and emotional support [20], financial problems [20, 21], physical limitations [20], multiple problems with medications [20], and lack of knowledge [22]. Also, compared to adults, adolescents may have limited abilities to have health-promoting behaviors or may not consider themselves vulnerable to the long-term consequences of diseases [23]. However, some factors related to health-promoting behaviors may vary within different communities and cultures, for which a comprehensive summary is necessary. Therefore, the current scoping review provides a complete picture of related factors to develop interventions for encouraging adolescents’ health-promoting behaviors. This review doesn’t aim to produce a critically appraised and synthesized result but rather to provide an overview or map of the evidence in the health-promoting behaviors in adolescents; hence, a scoping review was the most appropriate methodology [24]. The current study’s findings can help encourage health-promoting programs in adolescents in Iran and other developing countries by reviewing the influencing factors in health-promoting behaviors among adolescents.

## Methods

The latest JBI guidance for scoping reviews was used to conduct this scoping review [25]. The general purpose of conducting this scoping review is to map and identify the available evidence to address an exploratory research question. We used the PRISMA-ScR (Preferred Reporting Items for Systematic reviews and Meta-Analyses extension for Scoping Reviews) checklist to report our results (Additional file 1) [26].

### Step 1. Identifying the research question

In the first step of this scoping review, we sought to answer the following question: Overarching question:

Which factors affect health-promoting behaviors in adolescents in the literature?

### Step 2. Search strategy

In this scoping review, the relevant search was conducted using the identified keywords and their related words in databases, including PubMed, Web of Science, Science Direct, ProQuest, and Scopus (See Table 1). Keywords were searched in the title and abstract of papers via an advanced database search. All reference lists were reviewed to identify relevant studies that our database searches could have missed. Forward and backward searches were also done. The article’s publication time was limited to 1977–2020.

**Table 1:** Search terms in english

Database	Search strategy
**PubMed**	(adolescent[Title/Abstract] OR teenage*[Title/Abstract] OR youth[Title/Abstract]) AND “self-care“[Title/Abstract] OR “health promotion" [Title/Abstract]
**Science direct**	TITLE-ABSTR-KEY (adolescent OR teenage* OR youth) AND TITLE-ABSTR-KEY (“self -care” OR “health promotion" )
**Scopus**	#1: TITLE-ABS-KEY ( "self-care" ) #2:TITLE-ABS-KEY ( teenage* ) OR TITLE-ABS-KEY ( adolescent ) OR TITLE-ABS-KEY ( youth ) #1 AND #2 Search within: ( ALL ( "health promotion" ) )

### Step 3. Inclusion and exclusion criteria

All peer-reviewed empirical/original studies that described health-promoting behaviors among healthy adolescents were eligible to be included in the review. Also, studies written in the English language were included in the review. Articles that described health-promoting behaviors among adolescents with diseases were excluded. Furthermore, papers presenting systematic / scoping / rapid review results were excluded from the data extraction process.

### Step 4. Evidence screening and selection

**Fig. 1:**
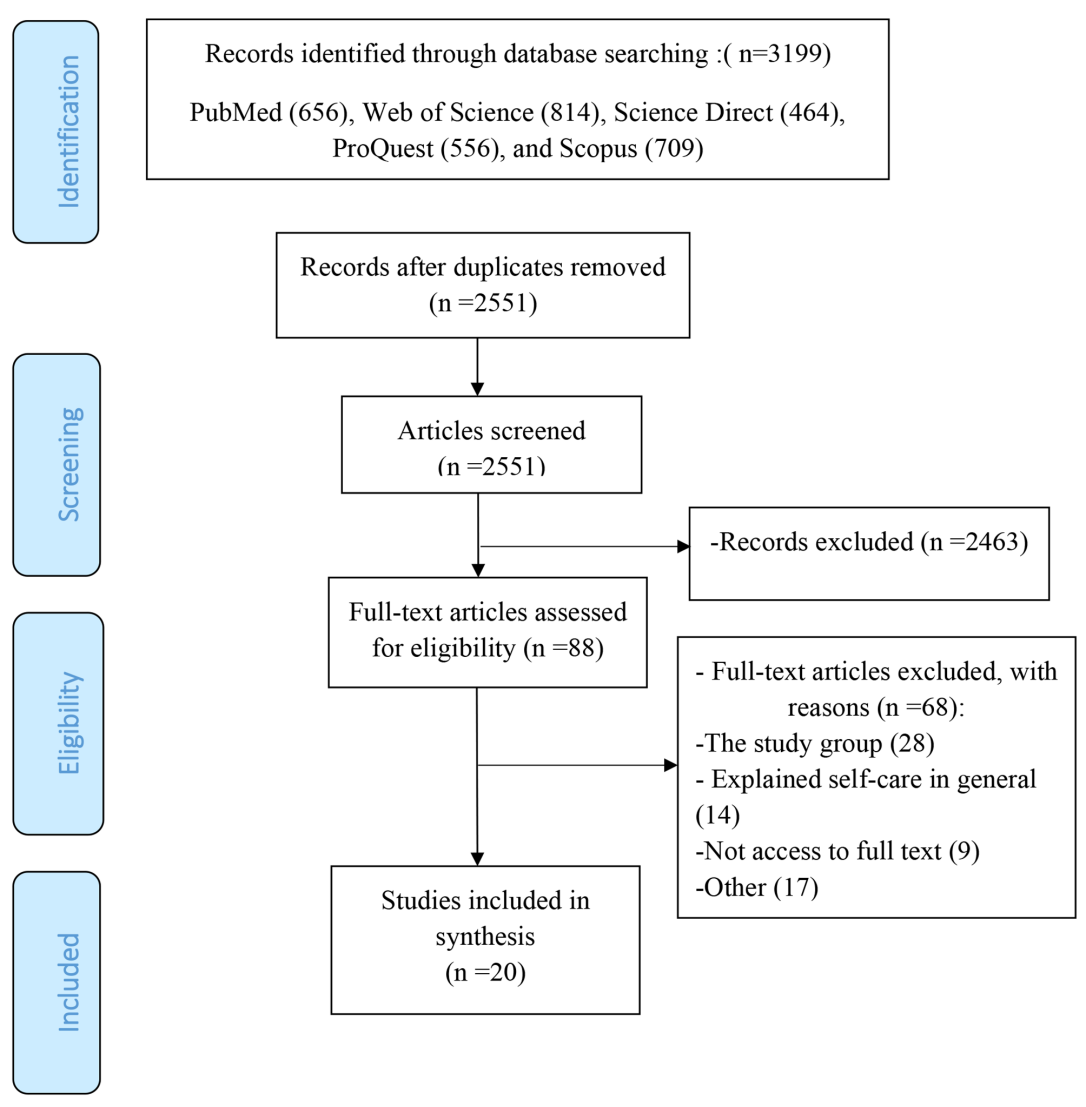
Flow chart explaining the selection of primary study

All references retrieved through the initial search were saved in an EndNote® library (9.3) and reviewed for relevance. In the first phase, the titles and abstracts of the articles were independently evaluated by two reviewers for inclusion in the study, and those irrelevant to the topic of the review were excluded. We then independently reviewed the full papers for inclusion. In the next phase, two researchers retrieved and reviewed full texts (NK, LD). Disagreements over study selection and data extraction were resolved by consensus and discussion with other reviewers to make a final decision on inclusion if necessary. The study selection process is presented in Fig. 1. The two reviewers (RK and NK) independently appraised the quality of the included studies using the Critical Appraisal Skills Program (CASP) developed for different designs, including ten questions on the purpose of the study, appropriateness of the methodology, research design, data collection, and data analysis [27]. Any disagreements between reviewers in this phase were resolved by the contribution of a third reviewer (LD).

### Step 5. Data extraction

Two researchers conducted data extraction using a standardized data collection form. First, a summary of each study, including the name of the first author, publication year, study setting (countries of origin), participants, sample size, and factors affecting health-promoting behaviors were extracted, and any disagreements were resolved by discussion, and if this failed, a resolution was reached through a third researcher (NK) to make a final decision.

### Step 6. Data analysis

The researchers utilized inductive synthesis to examine the data and identify the factors affecting promoting healthy adolescent behaviors. Two investigators, NK and LD, independently coded and categorized the influential factors. In case of any disagreements, the researchers resolved them through discussion, and if they failed to reach a consensus, a third researcher contributed to the resolution process.

## Results

The initial search yielded 3199 citations. Full texts were retrieved for 88 potentially eligible studies. After exclusion, 20 studies were included in the review (Fig. 1). The main characteristics of the included studies are displayed in Table 2. Most studies used a cross-sectional design (n = 14; 70%); the rest used longitudinal, randomized controlled, retrospective and quasi-experimental designs. Half of the included studies were from North America, including the United States and Canada (n = 11; 55%). Five studies originated from Asia, and the rest were conducted in European counties. In the overall assessment, the methodological quality of 16 reviewed studies (80.0%) was rated as ‘High’, while 4 (20%) studies were rated as ‘Medium’.

**Table 2:** Characteristics of validation studies in the review and summary of results

Author/ Year	Country	Study design	Participation	Sample size	Quality appraisal	Affecting Factors
Armitage (2013) [28]	Romania	Randomized Exploratory	High school students 13–19 year old	238	High	Self-incentive (reward), economic incentives
Callaghan, (2006) [29]	US	Descriptive and inferential	Adolescents aged 14 to 19	256	High	Support system, Adequate income, Gender, Adequate living conditions, Church attendance, Medical problems/disabilities, Hope, Self –concept, Self-esteem
Callaghan (2005) [30]	US	Descriptive multivariate	Adolescents aged 14 to 19	256	High	Responsibility, initiative
Chen et al., (2005) [31]	Taiwan and US	Cross-sectional Descriptive	Adolescents aged 12–15 years	550	High	School health promotion programs, National health insurance, Parental socioeconomic status, Cultural factors
Ergun et al., (2011) [1]	Turkey	Quasi-experimental	Sixth-grade students	58	High	Individualized education, Setting and Environment, Positive communication between adolescents and their mothers, Mothers’ knowledge level
Haddad et al., (2009) [32]	Jordan	Cross-sectional	Students 12–17 year old	530	High	Ease of access, Media messages, Commercials, School-based health promotion
Jessor, et al., (1998) [33]	US	Longitudinal	7th-, 8th-, and 9th-grade adolescents	1,493	High	Proximal protective factors (Value on health, Perceived effects of health-compromising behavior, Parents who model health behavior, Positive relationships With adults) and Distal protective factors(A commitment to school, Having friends who take part in conventional activities like youth groups and community volunteer work, Involvement in prosocial activities, and Church attendance, Religiosity)
McCaleb et al., (2000) [34]	US	Descriptive and inferential	15- and 16-year-old adolescents	425	Medium	Sociocultural characteristics, Church attendance, Health education programs
Melnyk et al., (2009) [35]	US	A Randomized Controlled Pilot	Hispanic adolescents, ages 14–16 years	19	High	COPE (Creating Opportunities for Personal Empowerment)
Canty-Mitchell (1993) [36]	US	Descriptive correlational	Adolescents from 13 to 19 years of age	202	High	Hope and type of school
Uzuncakmak, et al., (2017) [37]	Turkey	Quasi-experimental	Adolescents 13 years of age	30	High	Education, Parents’ attitude, Mothers’ and fathers’ education level parents’ age group, Family democratic approach
Bakouei et al., (2018) [38]	Iran	Cross-sectional	College Students	350	Medium	Self-efficacy, Gender, Family size, Living in dormitory
Almutair et al., (2018) [39]	Saudi Arabia	Descriptive cross-sectional	University students	1,656	Medium	Family structure, Gender, Type of college, Year in school
Gillis (1994) [40]	Canada	Descriptive correlational	Female adolescents and their parents	184	High	Perceived self-efficacy, Perceived health status, Ethnicity
Molaifard et al., (2020) [41]	Iran	Cross-sectional	High school students	400	Medium	Motivation skills constructs, Behavioral skill construct
Rice et al., (2019) [42]	US	Descriptive cross-sectional	Adolescents from 12 to 17 years of age	1,859	High	Perceived peer norms
Lee et al., (2019) [43]	Hong Kong	Retrospective correlation	Primary 4 (aged 9 to 10 years) and Secondary 3 (aged 14 to 15 years) students	54	High	Action competencies, Community link, Physical environment, Social environment, Healthy school policies, Services of school health protection
Haidar et al., (2019) [44]	US	Cross-sectional	High school students (8th and 11th grade students)	6,716	High	Parental physical activity support, Peer physical activity support, Parental social support, Peer social support, Gender
Xiao et al., (2019) [45]	US	Cross-sectional	Adolescents from 12 to 18 years of age	14,506	High	Physically activity, Computer use, Intake of fruits/vegetables in diet
Ozturk et al., (2020) [46]	Turkey	Cross sectional and correlational	Secondary school students (6th, 7th and 8th grade students)	2,498	High	Income level, Father education level, Mother education level, Grade

Table 3 presents factors affecting health-promoting behaviors extracted in the included studies. These factors were categorized into six main items, including educational, economic, socio-cultural, spiritual, psychological and personal.

### Economic factors

Economic factors such as providing incentives and coverage of the national health insurance were influential factors in promoting healthy behaviors in adolescents [28–30, 36, 47]. The results showed that adolescents residing in higher-income areas or being in middle-class or higher-income families were most likely to involve in healthy behaviors [48]. Adolescents apprehending that they had the monetary resources to meet daily needs practiced healthy behaviors more frequently, had higher levels of self-efficacy, and had more self-care abilities [29]. In addition, high levels of fear and anxiety, substance use, deviance, aggression, misconduct, and low school attendance and low academic performances were related with poor conditions in families [48].

### Educational factors

Several studies indicated that educational interventions effectively promote healthy behaviors in adolescents [32, 34, 35, 37, 41, 47, 49–51]. Planned self-care education for adolescents helps them to develop self-care attitudes [37]. Schools were the most cited settings to provide health-promoting education among adolescents [32, 47]. The level of parents’ education was identified as an important educational factor [34, 37, 49]. Health-promoting behaviors were improved in adolescents by increasing parents’ education levels [34, 37, 49]. Adolescents with parents with lower educational levels practiced less healthy behaviors [47]. Educational factors were among the most important factors in health-promoting behaviors in adolescents [32, 34, 35, 37, 47, 49–51].

### Social factors

Social factors included support systems, peers, and responsibility [29, 30, 50]. A support system including family, friends, teachers, neighbors, healthcare providers and clergies affected healthy behaviors among adolescents Adolescents with stronger support systems practiced health-promoting behaviors more frequently and had higher levels of self-efficacy and more skills in healthy self-care behaviors [29, 38]. One study showed a significant effect of peers on adolescents’ behaviors [50]. Teenagers spend more time with their friends, and peer interactions generally take place farther from home and without direct parental supervision [52]. Peer pressure can result in high-risk behaviors during adolescence. For instance, the consumption of cigarettes and drugs as a result of peer encouragement and poor school achievements [50, 52] can be observed among adolescents.

### Spiritual factors

Our review showed that spiritual factors are among main factors that can influence health behaviors among the adolescents [34, 47, 53]. Strong spiritual beliefs may lead to a more purposeful and healthier lifestyle [50]. Church attendance was positively correlated with self-care [34]. The adolescents who routinely practiced a religion also practiced health promoting behaviors more frequently and had higher self-efficacy [34].

### Psychological factors

Psychological factors were also among the influencing factors in adolescents’ health-promoting behaviors [28, 30, 36, 47–50]. A personal sense of confidence and control could enhance health behavior and produce positive results regarding healthy habits, recovery from illness and any chronic health condition [54]. Another influential psychological factor in our review was self-incentive (reward), which had a strong potential for changing health behavior [28]. Likewise, children’s initiative in decision-making led to more appropriate behavior in health care [30, 50, 55]. Students aiding the school nurse in schools’ health decisions had more self-confidence in their health management [55] and adolescents who communicated with their mothers more and had positive relationships with their friends and parents had better health-promoting behaviors [49, 50].

### Personal factors

Our review showed that some personal factors such as family structure, life patterns, gender, and medical problems correlated with health behavior [29, 37, 47]. Adolescents living in families with a democratic style of childbearing benefited from more self-care education [37]. Also, adolescents living with both parents were considered more self-care in terms of health-related behaviors in comparison to those living with single parents [31]. Gender was another factor affecting healthy behaviors [29]. The results of studies on the role of gender in health-promoting behaviors were not consistent. Some studies showed that females had more positive health behaviors than males [47, 49]; while in a number of other studies, health-promoting behaviors were higher in male adolescents [29, 37].

**Table 3:** Effective factors in health promoting self-care behaviors in adolescents

Themes	Codes
Personal factors	1-Gender, 2-Family structure, 3-Medical problems
Psychological factors	1-Self-incentive(reward), 2-Self –concept, 3-Self-esteem, 4-Initiative, 5-Positive communication
Spiritual factors	1-Church attendance 2-Value on health, 3-Religiosity
Sociocultural factors	1-Support system, 2- Responsibility, 3- Peer support
Economic factors	1-Economic incentives, 2-Income, 3-National health insurance
Educational factors	1-Health education programs, 2-Parents’ education level, 3 -Perceived effects of health

## Discussion

This scoping review aimed to identify and synthesize factors influencing health-promoting behaviors in adolescents. We identified various influential factors on health-promoting behaviors in adolescents. Majority of the included studies emphasized on the role of education in health promoting behaviors [32, 34, 35, 47, 49]. Several studies showed that adolescents’ self-care skills are another crucial factor [37, 49, 51].

Educational programs improved healthy behaviors, reduced morbidities and utilization of healthcare resources. They led to reduced absenteeism from school, the number of days with restricted activity, and a number of visits to an emergency department. Like a domino effect, students receiving health-promoting educations will positively influence their families and friends [56]. The overall benefit is transmission of the skills to new generations when these young people become parents. Interactions between teachers, parents, and students are essential to promoting healthy behaviors in adolescents [29, 47]. Teachers’ participation in health education programs in schools may influence students’ health promoting behaviors. Moreover, providing health education to students through peers can improve adolescents’ knowledge, attitude and behavior and may increase their health responsibility [57].

In our review, economic factors had an important role in improving health promoting behaviors in adolescents [29, 47]. Adequate income, adequate living conditions and parental socioeconomic status positively affect health. Different economic and cultural interventions have been developed to promote healthy behaviors [58–60]. Economic discrimination against adolescents can have adverse consequences in adulthood [61]. Social and economic welfare and equity can provide a healthy future for adolescents.

The support system was identified as an influential factor in adolescents’ healthy behaviors, self-efficacy, and self-care [29]. High school students with support systems often have healthy behaviors and high self-efficacy and self-care abilities. Knowledge of and access to social support systems within the community can play an essential role in identifying and meeting adolescents’ health needs [29, 62, 63].

Our review showed that health responsibility did not play an important role in prompting healthy behaviors. This can be due to lower age and less experience of adolescents [30, 64, 65]. People are usually only aware of their health disorders once they face a health issue, as most teenagers have not experienced serious health problems, their health awareness is less than that of adults, and they are irresponsible for their health. School educational programs can increase health responsibility and positive attitudes towards health and healthy behaviors [39, 66, 67].

Proper communication and having friends who take part in conventional activities like youth groups and community volunteer works were some ways to improve adolescents’ health [50]. Adolescents’ participation in school activities and activating the network of student volunteers can be effective in promoting social health and health responsibility. Interpersonal relations and social support were among the most cited health indicators in different studies [47, 68] and existed while people could make relations with others continuously. Interpersonal relationships among adolescents should be improved through proper educational interventions such as providing workshops on life skills and the effective communication.

Religious beliefs and church attendance positively affected health behaviors [29, 34, 50]. One study showed that religious beliefs had a protective role in relieving mental disorders such as anxiety, depression, addiction, suicide and sexual deviations [69]. Self-esteem is a main component of behaving healthily [29]. People with high levels of self-esteem can create positive changes in one’s personal and social lives. The results showed that adolescents with higher self-esteem had healthier behaviors [29]. In addition, self-incentives are potentially powerful means of changing health behavior [28]. Research studies have indicated that mothers can affect adolescents’ self-care levels. Our review showed that mothers’ communication with adolescents could affect their self-efficacy. Health behaviors were improved in adolescents by increasing mothers’ awareness and the relationship between mothers and adolescents [1].

Family is a main factor in developing adolescent behavior [29, 37]. Family shapes individuals’ physical and mental health and depends on the relationship between individuals and their families. In appropriate and healthy families, adolescents can freely discuss their problems and consult with family members to find solutions to their problems. Adolescents who are not emotionally and securely assisted in their families suffer from a variety of hardships, such as drug use, violence, and delinquency. Developing proper interventions can increase families’ knowledge about parenting styles and communication skills to improve adolescent health through making good relationships with them. For future research, it is suggested to develop research based on intervention to improve adolescent health-promoting behaviors. It is recommended that each factor affecting health-promoting behavior, such as educational, economic, social, spiritual, psychological and personal factors, be examined separately, in detail, and in-depth. Also, it will be effective to develop programs in social networks that can inform people about health programs, sensitize them to their health status and provide strategies to improve health-promoting behaviors.

### Limitations of the study

A comprehensive approach was taken to exploring a variety of sources to synthesize what is known about factors affecting health-promoting behaviors among adolescents. Including steps such as having two reviewers for every complete source and developing a search strategy in consultation with a librarian have added rigor to the exploring process and thus serve as strengths. However, this review had two limitations. First, we only searched in five electronic databases. As a result, some evidence might have been excluded. The second is that only English-language sources were retrieved and reviewed—no doubt there is literature on health-promoting behaviors produced in other languages.

## Conclusion

Health-promoting behaviors among adolescents require careful consideration. Our review showed that various educational, economic, social, spiritual and personal factors affect health-promoting behaviors in adolescents. All of these factors are important in shaping behavior. Based on the study findings, it is recommended that policymakers and healthcare providers develop several interventions based on identified factors to empower adolescents to perform health-promoting behaviors.

### Electronic supplementary material

Below is the link to the electronic supplementary material.

Supplementary Material 1

## Data Availability

All data generated or analysed during this study are included in this published article.

## Abbreviations

NCDs: Non-communicable diseases
WHO: World Health Organization
CASP: Critical Appraisal Skills Program
